# Awareness of cervical cancer risk factors and symptoms: cross‐sectional community survey in post‐conflict northern Uganda

**DOI:** 10.1111/hex.12382

**Published:** 2015-07-23

**Authors:** Amos D. Mwaka, Christopher G. Orach, Edward M. Were, Georgios Lyratzopoulos, Henry Wabinga, Martin Roland

**Affiliations:** ^1^ Department of MedicineSchool of MedicineCollege of Health SciencesMakerere UniversityKampalaUganda; ^2^ Department of Community Health and Behavioral SciencesSchool of Public HealthCollege of Health SciencesMakerere UniversityKampalaUganda; ^3^ Management Science for HealthKampalaUganda; ^4^ Department of Health Services ResearchInstitute of Public HealthUniversity of CambridgeCambridgeUK; ^5^ Kampala Cancer RegistryDepartment of PathologyCollege of Health SciencesMakerere UniversityKampalaUganda

**Keywords:** awareness, cervical cancer, health seeking, perceived causes, post‐conflict northern Uganda, risk factors

## Abstract

**Background:**

Lack of awareness of risk factors and symptoms for cancer may lead to late diagnosis and poor prognosis.

**Objective:**

We assessed community awareness about cervical cancer risk factors and symptoms and perceptions about prevention and cure of cervical cancer in order to contribute data to inform interventions to improve cervical cancer survival.

**Design:**

Cross‐sectional population‐based survey.

**Setting and participants:**

We conducted this study in Gulu, a post‐conflict district in Uganda in 2012. The sample included 448 persons aged 18 years and above, selected through a multi‐stage stratified cluster sampling process.

**Data collection methods and analysis:**

We collected data using a pretested structured questionnaire. Logistic regressions were used to determine magnitudes of associations between socio‐demographic and outcome variables.

**Results:**

Most participants (444/448) had heard about cervical cancer. Known risk factors including multiple sexual partners, human papillomavirus infection, and early onset of sexual activity, were recognized by 88%, 82%, and 78% of respondents respectively. 63% of participants believed that prolonged use of family planning pills and injections caused cervical cancer. The majority of participants recognized symptoms of cervical cancer including inter‐menstrual bleeding (85%), post‐menopausal bleeding (84%), and offensive vaginal discharge (83%). 70% of participants believed that cervical cancer is preventable and 92% believed that it could be cured if diagnosed at an early stage.

**Discussion and conclusions:**

Recognition of cervical cancer risk factors and symptoms was high among study participants. Targeted interventions including increasing availability of HPV vaccination, population‐based cervical screening and diagnostic services can translate high awareness into actual benefits.

## Background

Cervical cancer is a leading cause of morbidity and mortality among women in the low‐ and middle‐income countries (LMICs). Of 500 000 new cervical cancer cases diagnosed annually worldwide, 83% occurred in LMICs where more than 80% are diagnosed at advanced stage and have poor treatment outcomes.^1^, ^2^, ^3^

The reasons for the high incidence and mortality from cervical cancer in sub‐Saharan Africa include lack of awareness of cervical cancer among the population, health‐care providers and policymakers; limited access to high‐quality health‐care services and cervical screening programmes; and lack of functional referral systems. All these lead to advanced stage at diagnoses.^4^ In developed countries, incidence and mortality from cervical cancer have been reduced through measures which include cytological screening and prompt treatment of early cervical lesions.^1^, ^5^ Introducing population‐based cervical screening and increasing its uptake in the LMICs is an important goal in reducing cervical cancer mortality. Uptake of screening programmes may be assisted by raising awareness about cervical cancer risk factors including young age at first sexual intercourse,^6^, ^7^ multiple male sexual partners,^8^ high parity,^9^, ^10^ infections with the human papillomavirus,^11^, ^12^ young age at first full‐term pregnancy,^13^ prolonged use of oral contraceptives^14^, ^15^, ^16^ and HIV infections.^17^, ^18^ Similarly, early help‐seeking may be promoted if women in the LMICs become more aware of the symptoms of cervical cancer including intermenstrual vaginal bleeding, post‐menopausal vaginal bleeding, post‐coital vaginal bleeding, offensive vaginal discharge and lower abdominal pain.^19^, ^20^, ^21^

There is evidence that raising awareness of cancer symptoms and signs might increase people's ability to detect early symptoms and signs of cancer.^22^ In the United Kingdom (UK), knowledge and understanding of cancer risk factors and outcomes of cancer treatments influenced individuals' intentions and actual participation in cancer prevention programmes.^23^ In addition, better knowledge of cancer warning signs has also been linked with early help‐seeking.^24^ Regarding cervical cancer, a substantial body of research has shown that awareness of cervical cancer and knowledge of its risk factors and symptoms can increase uptake of cervical screening and encourage early help‐seeking for symptoms suggestive of cervical cancer.^4^, ^25^ In an educational intervention in India, increasing awareness about cervical cancer symptoms to 76% in an intervention community as compared with 25% in the control community was associated with significant change in stages at presentation of cervical cancer from 38% in stages I and II before the intervention to 51% in stages I and II after the intervention.^26^

In Uganda, there are limited data on levels of population awareness about cervical cancer, including perceptions, beliefs and knowledge about cervical cancer risk factors and symptoms. The objective of this study was to investigate community awareness about cervical cancer risk factors and symptoms and beliefs about cervical cancer prevention and treatment outcomes in post‐conflict northern Uganda. Findings from this study may inform policies relating to cervical cancer in rural and post‐conflict regions and lead to reduction in deaths from cervical cancer.

## Methods

### Study design and setting

We conducted a population‐based cross‐sectional survey in two of three parliamentary constituencies in Gulu district during September and October 2012; the three parliamentary constituencies include the Municipality, Aswa county and Omoro county with 53, 82 and 148 villages, respectively. Between 1987 to 2006, more than 90% of the rural population of Gulu district and the mid‐northern region was forced into internally displaced peoples (IDP) camps due to a civil conflict between the government of Uganda and the Lord's Resistance Army (LRA) rebels.^27^ In the IDP camps, there was a high incidence of rape and prostitution which increased vulnerability to HIV^28^ and perhaps HPV infections which in turn could increase predisposition to cervical cancer.

### Sample size

Sample size was calculated using Kish Leslie formula^29^; the prevalence of awareness about cervical cancer risk factors and symptoms was estimated at 50% because no studies were found in literature for similar population. The calculated study sample size of 384 was based on the estimated 50% prevalence, a precision of 5% and to allow a 95% interval around estimates. Of the 384, 15% of participants^30^ were to be recruited from the municipality and 85% (326) from Aswa, a rural county. To increase statistical power to identify yet unknown characteristics of the population related to awareness of risk factors and symptoms of cervical cancer, we oversampled participants from both study sites; 354 from Aswa County and 94 from Gulu municipality.

### Sampling procedure

We purposefully selected Gulu municipality; Aswa was randomly sampled from two rural counties – Aswa and Omoro. Computer‐generated random number digits were then used to randomly select 10 of 53 villages from the Municipality and 33 of 82 villages in Aswa. In this study, each selected village was considered a cluster and from each cluster, we randomly selected 10 households and interviewed one participant per household. A household was defined as a dwelling place with a group of people including parents, guardians and children who share food from one cooking place. A homestead was defined as a set of households in one compound. In homesteads where there were three or more households, two households were sampled.

In each village, the central point of the habitable area of the village was located; at this point, a research assistant spanned a pen to determine the direction along which households were sampled. The direction where the tip of the pen pointed was chosen. A list of five households was generated, and one household was selected by simple random approach as the starting household. Subsequently, every third household was selected until 10 households were sampled. This sampling approach is a modification of the expanded program on immunization (EPI) sampling method (30 clusters × 7 households) that has been used in Africa and other countries where household lists are lacking^31^.

### Selection of study participants

Community members aged 18 years and above were included. We excluded people with documented or self‐reported hospital diagnosis of cervical cancer, and those whose daughters or wives had diagnoses of cervical cancer (reported or documented). Recruitment was conducted between 1.00 and 6.30 PM when prospective participants were expected to be back home from the gardens. When no adults were found in a selected household, the immediate next household was selected. In each selected household, verbal consent to participate in the study was sought before generating a list of all eligible participants in the household. From the list, a simple random sampling approach was used to select one participant from the household for the interview. The objectives of the study, and potential benefits and harms were read to the selected participants before soliciting written informed consents. Research assistants interviewed study participants in quiet and convenient locations within the homesteads where interruptions were minimal and no non‐participants were present.

### Data collection procedure

We collected data using a structured pre‐tested questionnaire which contained items adapted from findings and tools used in studies in East Africa^32^, ^33^, ^34^ and South Africa.^35^, ^36^ The questionnaire included perceived cervical cancer risk factors reported by participants from the same community during a qualitative focus group discussion that preceded this survey. Interviews were conducted in Acholi – the major Uganda language spoken in Gulu. The questionnaire was designed in English and translated into Acholi language by two natives conversant in both English and the local language. The tool was back translated into English by a pair of translators not familiar with the original version; the two versions were compared for conceptual equivalence and harmonized. A final translation into Acholi was then performed and checked for accuracy and preservation of meanings.

Research assistants (RAs) included three men and three women with bachelor degrees in social sciences and education. The RAs were trained for 2 days on principles of quantitative and survey research including data collection, on the objectives of this research, sampling procedures, interview techniques and consent procedures. Pre‐testing of the tools was performed with 5 men and 5 women in the nearby community. The questionnaires were administered by three pairs of research assistants including a man in each pair. Each pair collected data from 10 to 12 participants in a village per day. Each research assistant interviewed participants independently. The principal investigator reviewed the data collected daily to ensure quality and comparability of data between research assistants. Data collection lasted 18 days.

### Data management and analysis

Two independent clerks entered data using Epidata 3.1 (EpiData Software, Odense, Denmark). EMW synchronized and cleaned the data files and then exported data to STATA I/C version 12 (StataCorp LP, College Station, TX, USA) for analysis. Descriptive statistics including means, standard deviations and proportions were generated. Chi‐square tests were used to determine associations between independent variables including socio‐demographic characteristics and dependent variables including awareness and perceptions of cervical cancer risk factors and symptoms. Logistic regression models were used to determine the magnitudes of associations between independent and dependent variables; odds ratios have been reported with accompanying 95% confidence intervals.

### Ethical approval

This study was approved by Makerere University School of Medicine Research and Ethics Committee (SOMREC) and the Uganda National Council of Science and Technology (UNCST). We obtained permission to access communities from the district and local leaders. Research assistants explained the purpose and procedure of the study to all participants and obtained individual written informed consents before conducting interviews. Participants were informed of their freedom to decline participation if they chose to and or join study and withdraw at any point without fear of retribution from the study team. After the interviews, about 5–10 min was allowed for participants to ask questions related to cervical cancer. A modest amount of money for refreshments during the question and answer sessions was provided to participants.

## Results

Of 456 prospective participants approached, two women aged about 70 years declined to participate; these two women chose to provide no reasons for non‐participation. Six questionnaires were incomplete (missing data on any three of residence, age, gender, number of biological children and level of education) and were excluded from analysis, leaving 448 for analysis. The majority of participants (75.7%) were women. More than two‐thirds of participants (77.2%) either had no formal education or had only primary school education. In total, 93.1% of participants were not formally employed; women were less likely to be formally employed compared with men (*P* = 0.007). In total, 71.6% of participants were married (Table 1).

**Table 1:** Socio‐demographic characteristics of study participants

Demographic characteristics	Sex		Total population responding	*P*‐values
	Male, *n* (%)	Female, *n* (%)		
Residence				
Aswa county	87 (79.8)	267 (78.8)	354	0.814
Municipality	22 (20.2)	72 (21.2)	94	
Age groups (years)				
18–29	37 (33.9)	135 (39.9)	172	0.09
30–44	41 (37.6)	116 (34.3)	157	
45–59	14 (12.8)	57 (16.9)	71	
≥60	17 (15.6)	30 (8.9)	47	
Missing		1	1*	
Mean age (±SD)	38.3 ± 15.0	36.4 ± 14.3	36.8 ± 14.4	
Religion				
Catholic	86 (79.6)	258 (76.3)	344	0.783
Anglican	16 (14.8)	51 (15.1)	67	
Muslim	1 (1.0)	5 (1.5)	6	
Pentecostals/born again	5 (4.6)	24 (7.1)	29	
Missing	1		1	
Marital status				
Never married	13 (11.9)	10 (2.9)	23	**<0.001** **
Married	85 (78.0)	236 (69.6)	321	
Others (divorced, widowed, widower)	11 (10.1)	93 (27.4)	104	
Age at first marriage (years)				
<18	8 (8.6)	151 (46.3)	159	***
18–29	79 (84.9)	175 (53.7)	254	
>30	6 (6.5)	0 (0.0)	6	
Missing	13	16	29	
Mean age at first marriage (SD)	23.2 ± 9.1	17.9 ± 2.6	19.0 ± 5.3	
Number of biological children				
None	12 (11.1)	21 (6.2)	33	0.148
1–4	47 (43.5)	156 (46.2)	203	
5–10	40 (37.0)	145 (42.9)	185	
≥11	9 (8.3)	16 (4.7)	25	
Missing	1	1	2	
Education attainment				
No formal education	2 (1.8)	84 (24.9)	86	**<0.001** **
Primary 1–7	42 (38.5)	175 (51.8)	217	
Completed primary education	17 (15.6)	25 (7.4)	42	
Secondary education (1–6)	33 (30.3)	41 (12.1)	66	
Tertiary and university	15 (13.8)	13 (3.8)	28	
Missing		1	1	
Employment status				
Student	5 (5.0)	6 (1.8)	10	0.007**
Formal employment	9 (8.9)	10 (3.0)	19	
Petty trader	14 (13.8)	33 (9.9)	43	
Peasant farmer	73 (72.3)	285 (85.3)	326	
Missing	8	5	13	
Total	109	339	448	

*Missing – not included in calculation of percentages, **Statistically significant ***chi‐square test not performed because one cell had zero value.

### Awareness about cervical cancer

Ninety nine per cent of participants (444/448) had heard about cervical cancer. Of these, 70.3% reported that cervical cancer is preventable while 92% reported that cervical cancer can be cured in the hospitals when diagnosed at an early stage. However, there was limited awareness that cervical cancer can be prevented through Pap smears (41%) or vaccination of young girls against HPV (8.3%). While about 1 in 3 (30.5%) participants had heard about the role of a sexually transmitted virus the development of cervical cancer, a larger proportion (85%) of participants believed that cervical cancer itself is sexually transmitted. Thine notion that cervical cancer spreads when a patient with cervical cancer has surgical treatment was reported by about 1 in 4 participants (29.9%) (Table 2). The odds of believing that cervical cancer spreads once operated on is 2.5 to 5 times higher among older participants (aged 45–59 and ≥60 years) compared to participants aged 18–29 years (Table S1a).

**Table 2:** Awareness and sources of information about cervical cancer

Domains inquired	Population, *n* (%)	Sex		Comparisons of male with female (reference)	
		Male, *n* (%)	Female, *n* (%)	Crude odds ratio	95% confidence interval
*Awareness about cervical cancer*					
1. Ever heard about cervical cancer					
Yes	444 (99.1)	108 (99.1)	336 (99.1)	0.96	0.10–9.39
No	4 (0.9)	1 (0.9)	3 (0.9)		
4. Cervical cancer is a sexually transmitted disease					
Yes	339 (85.0)	82 (86.3)	257 (84.5)	0.87	0.45–1.68
No	60 (15.0)	13 (13.7)	47 (15.5)		
5. Cervical cancer is preventable					
Yes	312 (70.3)	80 (74.1)	232 (69.0)	1.28	0.79–2.09
No	132 (29.7)	28 (25.9)	104 (31.0)		
6. Cervical cancer is preventable through vaccination of young girls					
Yes	37 (8.3)	9 (8.3)	28 (8.3)	1.00	0.46–2.19
No	407 (91.7)	99 (91.7)	308 (91.7)		
7. Cervical cancer is preventable through genital exams by health providers (Pap smears)					
Yes	182 (41.0)	44 (40.7)	138 (41.1)	0.99	0.63–1.53
No	262 (59.0)	64 (59.3)	198 (58.9)		
8. Cervical cancer is curable in hospitals when diagnosed early					
Yes	369 (92.0)	92 (88.2)	277 (99.1)	1.54	0.69–3.42
No	45 (8.0)	8 (11.8)	37 (0.9)		
9. Operation on patients with cervical cancer can spread cancer					
Yes	121 (29.9)	29 (28.2)	92 (30.5)	0.90	0.55–1.47
No	284 (70.1)	74 (71.8)	210 (69.5)		
*Common sources of information about cervical cancer*					
1. Radio					
Yes	307 (70.1) 307 (70.1)	84 (79.2)	223 (67.2)	1.87	**1.10–3.16** *
No	131 (29.9)	22 (20.8)	109 (32.8)		
2. Health personnel					
Yes	135 (31.5)	21 (21.0)	114 (34.8)	0.50	**0.29–0.85** *
No	293 (68.5)	79 (79.0)	214 (65.2)		
3. Family and friends					
Yes	82 (18.3)	23 (21.1)	59 (17.4)	1.27	0.74–2.18
No	366 (81.7)	86 (78.9)	280 (82.6)		

*Statistically significant.

The dominant sources of information about cervical cancer were the radio (70.1%), followed by health‐care professionals (31.5%) and then family and friends (18.3%). Men were significantly more likely to have heard about cervical cancer from the radio, unadjusted OR = 1.87 (95% CI; 1.10–3.16) but less likely than women to have heard from the health‐care professionals, unadjusted OR = 0.50 (95% CI; 0.29–0.85) (Table 2). On adjusting for gender, age, marital status, number of biological children and educational attainment, participants from the rural site were significantly less likely to hear about cervical cancer from the radio, adjusted OR (AOR) = 0.50 (95% CI; 0.27–0.93) Table S2a).

### Awareness about cervical cancer risk factors and symptoms

Participants were presented with a list and were asked to indicate which of the symptoms and risk factors listed were associated with cervical cancer. The majority of participants recognized known cervical cancer risk factors including multiple male sexual partners (88.3%) and cervical infection with a sexually transmitted germ or virus (82.0%). However, less than half of participants identified multiparity (48.6%) and cigarette or tobacco smoking (48.6%) as risk factors for cervical cancer (Table 3). There was no consistent association between recognition of cervical cancer risk factors and socio‐demographic characteristics including age, gender and residence of participants (Table S4a–d).

**Table 3:** Awareness about cervical cancer risk factors and symptoms

Recognition of	Responses			Total number of participants that responded
	Yes, *n* (%)	No, *n* (%)	Don't know, *n* (%)	
1. Cervical cancer risk factors				
Early onset of sexual activity	320 (78.2)	71 (17.4)	18 (4.4)	409
Infection with a sexually transmitted germ/virus (HPV)	342 (82.0)	51 (12.2)	24 (5.8)	417
Multiple male sexual partners	378 (88.3)	45 (10.5)	5 (1.2)	428
Smoking cigarettes/tobacco	203 (48.6)	182 (43.5)	33 (7.9)	418
Grand multiparity	195 (48.6)	181 (45.2)	25 (6.2)	401
2. Cervical cancer symptoms				
Intermenstrual vaginal bleeding	344 (84.5)	48 (11.8)	15 (3.7)	407
Post‐menopausal vaginal bleeding	354 (84.1)	48 (11.4)	19 (4.5)	421
Post‐coital vaginal bleeding	324 (76.1)	76 (17.8)	26 (6.1)	426
Excessive vaginal discharge, often with offensive smell	347 (83.0)	53 (12.7)	18 (4.3)	418
Lower abdominal pain	368 (87.6)	45 (10.7)	7 (1.7)	420
Pain in the genital during sexual intercourse (dyspareunia)	300 (71.1)	95 (22.5)	27 (6.6)	422

Recognition of cervical cancer symptoms was equally good; lower abdominal pain (87.6%) was the most frequently reported cervical cancer symptom. The other symptoms endorsed included intermenstrual vaginal bleeding (84.5%), post‐menopausal bleeding (84.1%), offensive vaginal discharge (83.0%), post‐coital vaginal bleeding (76.1%) and dyspareunia (71.1%) (Table 3).

### Perceived causes of cervical cancer

The questionnaire included perceived causes of cervical cancer reported in our previous focus group discussions in the region. Participants in this study reported causes of cervical cancer which included not washing women's genitals immediately after sexual intercourse (86.7%), sexual intercourse with polygamous men (83.9%), prolonged use of family planning pills and injections (63.3%), and engaging in rough sexual intercourse leading to physical trauma to women's genitals (68.5%) (Table 4). Other reported causes of cervical cancer included cultural issues and taboos such as engagement in sexual intercourse before marriage (52.4%) and annoying spirits of dead elders (13.1%).

**Table 4:** Perceived causes of cervical cancer

Perceived causes of cervical cancer	Responses			Total number of participants that responded
	Yes, *n* (%)	No, *n* (%)	Don't know, *n* (%)	
Family planning				
Use of family planning pills and injections	257 (63.3)	126 (31.0)	23 (5.7)	406
Act of sexual intercourse				
Cervical cancer is sexually transmitted	339 (76.7)	60 (13.6)	43 (9.7)	442
Sexual intercourse with a polygamous man	348 (83.9)	59 (14.2)	8 (0.9)	415
Sexual intercourse with a man who does not believe in God	205 (48.2)	200 (47.1)	20 (4.7)	425
Traumatic/rough sexual intercourse	281 (68.5)	113 (27.6)	16 (3.9)	410
Hygiene‐related perceptions				
Not washing the genitals well, especially after sexual intercourse	366 (86.7)	45 (10.7)	11 (2.6)	422
Sexual intercourse during menstrual period	253 (62.6)	119 (29.5)	32 (7.9)	404
Perceptions related to cultural beliefs and values				
Sexual intercourse before marriage	218 (52.4)	190 (45.7)	8 (1.9)	416
Cervical cancer is inheritable; you get it if your mother, aunt or grandmother had it	104 (25.5)	290 (71.1)	14 (3.4)	408
Cervical cancer affects a woman who annoys spirits of dead elders	56 (13.1)	364 (85.3)	7 (1.6)	427
Other perceived causes of cervical cancer				
Cervical cancer affects poor women	81 (20.2)	316 (78.8)	4 (1.0)	401
Cervical cancer is contagious; a woman gets it by being near a person with it	99 (23.7)	305 (73.1)	13 (3.1)	417

## Discussion

This study describes the perceptions, beliefs and awareness of the general population in Gulu about risk factors and symptoms of cervical cancer. It provides insights into community perspectives on whether cervical cancer is preventable and or curable. Most participants were aware of risk factors and symptoms of cervical cancer and believed that cervical cancer could be prevented and cured if diagnosed at an early stage. However, there was limited awareness about Pap smear and vaccination of young girls as preventive measures which may have been due to inadequate health messages about prevention or because screening services and vaccination against HPV are not widely available in the region. In addition, more than half of participants held certain beliefs about causes of cervical cancer which have the potential to make awareness campaigns on prevention less effective, and divert help‐seeking away from biomedical facilities. In this regard, some participants believed that cervical cancer develops when women defy cultural values prescribed by elders who have already died, women engage in sexual intercourse during menstrual periods and women do not wash their genitals after sexual intercourse. Globally, cultural beliefs, myths and stigmas about cancers are ubiquitous. Cultural beliefs have reduced Pap smear uptake and hampered health‐seeking for cervical cancer^37^, ^38^, ^39^ and require culture‐tailored interventions to reduce or eliminate them.^40^ Government programme managers and agencies in LMICs need to develop and adopt culturally sensitive approaches to dispel potentially harmful beliefs about cervical cancer. Furthermore, medical schools and other health training institutions need to develop and incorporate cultural competence courses into existing curricula so that health‐care professionals are capable of providing culturally competent care to their patients.^41^, ^42^, ^43^

A great proportion of participants also reported that cervical cancer can be prevented, treated and cured if diagnosed in early stage. This is consistent with findings in Ethiopia, where more than half of participants believed that cervical cancer can be prevented (63.5%), treated (66.1%) and cured if detected early (52.8%).^44^ High level of awareness about cervical cancer was also found in Democratic Republic of Congo (DRC)^45^ and South Africa.^46^ Limited awareness or inability to recognize cancer symptoms may lead to delay in presentations.^47^, ^48^ Awareness about risk factors and symptoms of cervical cancer have been found to be limited in most sub‐Saharan African countries including Kenya, Zimbabwe, Cameroon and Nigeria.^49^, ^50^, ^51^, ^52^ Differences in the characteristics of study population in the different studies including education, availability of health services and sources of information may explain in part some of the observed differences between studies. It is important not to lose sight of the fact that low awareness about cervical cancer risk factors, symptoms and Pap smears potentially precludes uptake of prevention^53^, ^54^, ^55^, ^56^, ^57^ and early health‐seeking.^47^, ^58^

Regarding HPV and its role in cervical cancer aetiology, awareness has varied across nations. In this study, no participant explicitly mentioned the HPV although 82% reported that a sexually transmitted virus may cause cervical cancer. Very limited awareness of the viral aetiology of cervical cancer and HPV vaccination has been observed in high‐income countries as recently as 2004^30^, ^59^ before the wider availability of HPV vaccination and supporting information campaigns when awareness improved.^60^, ^61^ The findings in Uganda are reminiscent of the limited awareness of these factors without public education efforts and in the absence of nationwide HPV vaccination. Governments in the LMICs and health development agencies need to make available population‐based HPV vaccinations alongside awareness campaigns about the role of HPV in the aetiology of cervical cancer. Otherwise prevention practices related to cervical HPV infections including vaccinations, delay of sexual activity and multiple male sexual partners may receive limited attention from the community.

The commonest source of information about cervical cancer in this study was the radio, particularly in urban setting and among men. In this regard, health education interventions to involve men and urban communities in promoting cervical cancer screening uptake and early help‐seeking could be channelled through radio talk shows. The second most common source was health‐care professionals, particularly among women. Perhaps women interact more with health‐care professionals during child and maternal health visits which men in East Africa rarely attend.^62^, ^63^, ^64^ In the DRC, the commonest source of information was health‐care professionals.^65^ These sources of information may all be used to increase awareness about HPV and Pap smears.

High parity as a risk factor for cervical cancer was underreported in this study and in other studies in sub‐Saharan Africa. In South Africa, only 44% of respondents identified high parity as a risk factor for cervical cancer^36^ while in Kenya, high parity and early onset of sexual activity were not reported at all.^66^ The underreporting of high parity in this study may relate to the fact that most of participants have experienced high parity themselves and would perhaps feel uncomfortable to perceive of and report high parity as a risk factor – a form of the self‐serving bias.^67^, ^68^, ^69^ On the other hand, underreporting high parity may be accounted for by some socio‐cultural beliefs favouring delivery of many children including the value of children as sources of wealth and security.^70^

In this study, participants reported other perceived causes of cervical cancer including prolonged use of hormonal contraceptives, not washing genitals following sexual intercourse and intercourse with polygamous men. These perceptions have the potential to reduce use of family planning methods but promote genital hygiene and minimize behaviour that would increase risk of cervical cancer. The perception about family planning was also reported in Kenya where participants thought that intrauterine devices cause wounds and infections in the uterus and eventually cause cervical cancer especially when poorly inserted.^66^ The majority of lay people seem to believe that prolonged use of family planning medicines can cause cervical cancer. Although a recent systematic review^71^ concluded that the evidence for increased risk of cervical cancer due to oral contraceptive use is insufficient, it is important to address the community concerns about the use of contraceptives in relation to increased risk of cervical cancer; otherwise, family planning methods might be avoided.

Regarding beliefs that sexual intercourse before marriage and with polygamous men can cause cervical cancer, abstinence until marriage will delay onset of sexual activity, delay exposure to high‐risk HPV infections at an early age and therefore reduce chances of developing cervical cancer, while avoiding sexual intercourse with polygamous men may reduce risk of contracting different strains of high‐risk HPV that lead to development of cervical cancer.^8^, ^72^ These beliefs provide fertile grounds for health policymakers and programme implementers to develop cervical cancer prevention campaigns. Campaigns should include husbands and local community leaders because they are essential in motivating women to participate in cervical screening programmes.^73^

Furthermore, a substantive proportion of participants held beliefs that cervical cancer is always fatal in spite of medical treatment, while others were unaware that cervical cancer is curable when diagnosed in an early stage. There is therefore need for targeted culture‐sensitive public campaigns to increase awareness about cervical cancer treatment and potential for cure when diagnosed in early stage, discourage fatalistic beliefs about cervical cancer and correct the belief that surgical operation leads to spread of cancer. Health messages could be channelled through health facility‐based talks as well as mass media including the local FM radios.

### Strengths and limitations

This study is a population‐based survey that involved both men and women. The study provides a benchmark for future evaluation of awareness campaigns and interventions to increase screening uptake and promote early health‐seeking for cervical cancer symptoms. A limitation to this study is the lack of a standard questionnaire for assessment of awareness of cervical cancer in sub‐Saharan Africa which limits our ability to compare results across studies. Second, the lack of equivalence for certain key words and phrases for example ‘cervical cancer development’ in the local language during translation could potentially alter meanings of questions and affect responses. We minimized loss of meaning during translation by adopting forward and backward translations by two pairs of independent translators who are proficient in both English and Acholi languages. In addition, the translators used local illness concepts gained during piloting of tools and from the preceding focus group discussions in the same communities.

## Conclusion

High awareness about cervical cancer, its risk factors and symptoms provides firm anchorage for targeted interventions to increase uptake of preventive methods and promote early health‐seeking for cervical cancer symptoms. Policymakers and health‐care systems in the LMICs need to make preventive methods available and easily accessed, including HPV vaccination and cervical screening, and facilities for early diagnosis and treatment of symptomatic cervical cancer so that awareness can be translated into actual benefits.

## Funding

The work was supported by Training Health Researchers into Vocational Excellence (THRiVE) in East Africa, Grant number 087540, funded by Wellcome Trust. The funding agency did not have any role in the design of this study, in data collection, analysis and interpretation; in the writing nor the decision to submit or where the manuscript be submitted.

## Conflict of interest

The authors declare that they have no conflict of interest.

## Supporting information


**Table S1** (a) Belief that cervical cancer spreads when surgical operation is done on the patient. (b) Belief that cervical cancer is a sexually transmitted disease (STD). (c) Belief that cervical cancer will always lead to death once it has affected a woman. (d) Belief that cervical cancer is curable only with traditional medicines.
**Table S2** (a) Have often heard about cervical cancer through the local FM radio. (b) Have often heard about cervical cancer from healthcare professionals.
**Table S3** (a) Awareness that cervical cancer is preventable. (b) Awareness that cervical cancer is curable in hospital when diagnosed in early stage.
**Table S4** (a) Recognized early onset of sexual intercourse as risk factor for cervical cancer. (b) Recognized multiple male sexual partners as risk factor for cervical cancer. (c) Recognized multi‐parity as risk factor for cervical cancer. (d) Recognized genital infection by a virus as risk factor for cervical cancer.
**Table S5** Reports contraceptives use as risk factor for cervical cancer.
