# Supplementary material for: Awareness of cervical cancer risk factors and symptoms: cross‐sectional community survey in post‐conflict northern Uganda
Source: Health Expect. 2015 Jul 23;19(4):854–67. doi: 10.1111/hex.12382 (PMC4957614; doi:10.1111/hex.12382)
Supplement: Supplementary file 1 — Table S1 (a) Belief that cervical cancer spreads when surgical operation is done on the patient. (b) Belief that cervical cancer is a sexually transmitted disease (STD). (c) Belief that cervical cancer will always lead to death once it has affected a woman. (d) Belief that cervical cancer is curable only with traditional medicines. Table S2 (a) Have often heard about cervical cancer through the local FM radio. (b) Have often heard about cervical cancer from healthcare professionals. Table S3 (a) Awareness that cervical cancer is preventable. (b) Awareness that cervical cancer is curable in hospital when diagnosed in early stage. Table S4 (a) Recognized early onset of sexual intercourse as risk factor for cervical cancer. (b) Recognized multiple male sexual partners as risk factor for cervical cancer. (c) Recognized multi‐parity as risk factor for cervical cancer. (d) Recognized genital infection by a virus as risk factor for cervical cancer. Table S5 Reports contraceptives use as risk factor for cervical cancer. [file HEX-19-854-s001.docx]

**Supplementary Data**

**Table S1 (a): Belief that cervical cancer spreads when surgical operation is done on the patient**

| **Patient demographic characteristics** | **Population responding** | **Cervical cancer spreads when operated on** | | **Crude OR (COR) (95% CI)** | **Adjusted OR (AOR) (95%CI)*** |
| --- | --- | --- | --- | --- | --- |
|  |  | **Yes** | **No** |  |  |
|  |  | **N (%)** | **N (%)** |  |  |
| **Residence** |  |  |  |  |  |
| Urban | 90 | 23 (19.0) | 67 (23.6) | 1.00 | 1.00 |
| Rural | 315 | 98 (81.0) | 217 (76.4) | 1.32 (0.77-2.24) | 1.22 (0.67-2.21) |
| **Sex** |  |  |  |  |  |
| Women | 302 | 92 (76.0) | 210 (73.9) | 1.00 | 1.00 |
| Men | 103 | 29 (24.0) | 74 (26.1) | 0.89 (0.55-1.47) | 0.81 (0.45-1.47) |
| **Age group (years)** |  |  |  |  |  |
| 18 - 29 | 160 | 41 (33.9) | 119 (42.0) | 1.00 | 1.00 |
| 30 - 44 | 143 | 34 (28.1) | 109 (38.5) | 0.91 (0.54-1.53) | 1.31 (0.66-2.61) |
| 45-59 | 63 | 25 (20.7) | 38 (13.4) | **1.91 (1.03-3.54)** | **2.50 (1.10-5.67)** |
| ≥ 60 | 38 | 21 (17.4) | 17 (6.0) | **3.59 (1.73-7.45)** | **5.21 (1.78-15.24)** |
| **Marital status** |  |  |  |  |  |
| Never Married | 22 | 5 (4.1) | 17 (6.0) | 1.00 | 1.00 |
| Married | 295 | 84 (69.4) | 211 (74.3) | 1.35 (0.48-3.79) | 2.32 (0.47-11.44) |
| Others | 88 | 32 (26.4) | 56 (19.7) | 1.94 (0.65-5.76) | 1.99 (0.35-11.38) |
| **Number of biological children** |  |  |  |  |  |
| None | 30 | 11 (9.1) | 19 (6.7) | 1.00 | 1.00 |
| 1 - 4 | 188 | 54 (44.6) | 134 (47.5) | 0.70 (0.31-1.56) | 0.37 (0.10-1.35) |
| 5 - 10 | 163 | 44 (36.4) | 119 (42.2) | 0.64 (0.28-1.45) | **0.20 (0.05-0.82)** |
| 11+ | 22 | 12 (9.9) | 10 (3.5) | 2.07 (0.68-6.36) | 0.49 (0.10-2.50) |
| **Education attainment** |  |  |  |  |  |
| No formal education | 67 | 23 (19.2) | 44 (15.5) | 1.00 | 1.00 |
| Primary education | 236 | 74 (61.7) | 162 (57.0) | 0.87 (0.49-1.55) | 1.10 (0.58-2.09) |
| Secondary education | 61 | 14 (11.7) | 47 (16.5) | 0.57 (0.26-1.24) | 0.80 (0.32-1.99) |
| High school | 12 | 2 (1.7) | 10 (3.5) | 0.38 (0.08-1.89) | 0.61 (0.12-3.42) |
| Tertiary/university | 28 | 7 (5.8) | 21 (7.4) | 0.64 (0.24-1.72) | 0.71 (0.23-2.19) |

*Adjusted for the socio-demographics on the table.

**Table S1 (b): Belief that cervical cancer is a sexually transmitted disease (STD)**

| **Patient socio- demographic characteristics** | **Population responding** | **Cervical cancer is an STD** | | **Crude OR (COR) (95% CI)** | **Adjusted OR (AOR) (95%CI)*** |
| --- | --- | --- | --- | --- | --- |
|  |  | **Yes** | **No** |  |  |
|  |  | **N (%)** | **N (%)** |  |  |
| **Residence** |  |  |  |  |  |
| Urban | 84 | 74 (21.8) | 13 (21.7) | 1.00 | 1.00 |
| Rural | 372 | 265 (78.2) | 47 (78.3) | 0.99 (0.51-1.93) | 0.68 (0.31-1.46) |
| **Sex** |  |  |  |  |  |
| Women | 304 | 257 (75.8) | 47 (78.3) | 1.00 | 1.00 |
| Men | 95 | 82 (24.2) | 13 (21.7) | 1.15 (0.59-2.24) | 1.30 (0.60-2.80) |
| **Age group (years)** |  |  |  |  |  |
| 18 - 29 | 158 | 121 (35.8) | 37 (61.7) | 1.00 | 1.00 |
| 30 - 44 | 138 | 126 (37.3) | 12(20.0) | **3.21 (1.60-6.45)** | **3.00 (1.19-7.60)** |
| 45 - 59 | 61 | 56 (16.6) | 5 (8.3) | **3.42 (1.28-9.18)** | **3.80 (1.09-13.22)** |
| ≥ 60 | 41 | 35(10.4) | 6(10.0) | 1.78 (0.70-4.57) | 2.34 (0.57-9.64) |
| **Marital status** |  |  |  |  |  |
| Never Married | 22 | 16 (4.7) | 6 (10.0) | 1.00 | 1.00 |
| Married | 285 | 242 (71.4) | 43 (71.7) | 2.11 (0.78-5.70) | 1.83(0.36-9.32) |
| Others | 92 | 81 (23.9) | 11 (18.3) | 2.76 (0.89-8.55) | 1.71 (0.27-11.00) |
| **Number of biological children** |  |  |  |  |  |
| None | 31 | 23 (6.8) | 8 (13.3) | 1.00 | 1.00 |
| 1 - 4 | 183 | 149 (44.2) | 34 (56.7) | 1.52 (0.63-3.70) | 1.74 (0.17-3.22) |
| 5 - 10 | 162 | 147 (43.6) | 15 (25.0) | **3.41 (1.30-8.94)** | 0.77 (0.15-4.06) |
| 11+ | 21 | 18 (5.3) | 3 (5.0) | 2.09 (0.48-9.02) | 0.42 (0.05-3.30) |
| **Education attainment** |  |  |  |  |  |
| No formal education | 73 | 64 (18.9) | 9 (15.3) | 1.00 | 1.00 |
| Primary education | 231 | 201 (59.3) | 30 (50.8) | 0.94 (0.42-2.09) | 1.27 (0.53-3.06) |
| Secondary education | 59 | 46 (13.6) | 13 (22.0) | 0.50 (0.20-1.26) | 0.74 (0.24-2.29) |
| High school | 12 | 10 (2.9) | 2 (3.4) | 0.70 (0.13-3.74) | 0.92 (0.14-5.90) |
| Tertiary/university | 23 | 18 (5.3) | 5 (8.5) | 0.51 (0.15-1.70) | 0.60 (0.15-2.42) |

*Adjusted for the socio-demographics on the table.

**Table S1 (c): Belief that cervical cancer will always lead to death once it has affected a woman**

| **Patient socio-demographic characteristics** | **Population responding** | **Cervical cancer always leads to death** | | **Crude OR (COR) (95% CI)** | **Adjusted OR (AOR) (95%CI)*** |
| --- | --- | --- | --- | --- | --- |
|  |  | **Yes** | **No** |  |  |
|  |  | **N (%)** | **N (%)** |  |  |
| **Residence** |  |  |  |  |  |
| Urban | 93 | 24 (20.5) | 69 (21.2) | 1.00 | 1.00 |
| Rural | 349 | 93 (79.5) | 256 (78.8) | 1.04 (0.62-1.76) | 0.83 (0.47-1.50) |
| **Sex** |  |  |  |  |  |
| Women | 335 | 88 (75.2) | 247 (76.0) | 1.00 | 1.00 |
| Men | 107 | 29 (24.8) | 78 (24.0) | 1.04 (0.64-1.70) | 1.38 (0.77-2.49) |
| **Age group (years)** |  |  |  |  |  |
| 18 - 29 | 170 | 39 (33.6) | 131 (40.3) | 1.00 | 1.00 |
| 30 - 44 | 155 | 38 (32.8) | 117 (36.0) | 1.09 (0.65-1.82) | 1.06 (0.53-2.10) |
| 45 - 59 | 70 | 20 (17.2) | 50 (15.4) | 1.34 (0.72-2.52) | 1.09 (0.47-2.49) |
| ≥ 60 | 46 | 19 (16.4) | 27 (8.3) | **2.36 (1.19-4.70)** | 1.42 (0.52-3.83) |
| **Marital status** |  |  |  |  |  |
| Never Married | 23 | 4 (3.4) | 19 (5.8) | 1.00 | 1.00 |
| Married | 316 | 81 (69.2) | 235 (72.3) | 1.64 (0.54-4.96) | **6.51 (1.23-34.35)** |
| Others | 103 | 32 (27.4) | 71 (21.8) | 2.14 (0.67-6.80) | **6.83 (1.12-41.46)** |
| **Number of biological children** |  |  |  |  |  |
| None | 33 | 12 (10.3) | 21 (6.5) | 1.00 | 1.00 |
| 1 - 4 | 200 | 47 (40.2) | 153 (47.4) | 0.54 (0.25-1.17) | **0.19 (0.05-0.68)** |
| 5 - 10 | 182 | 48 (41.0) | 134 (41.5) | 0.63 (0.29-1.37) | **0.17 (0.04-0.67)** |
| 11+ | 25 | 10 (8.5) | 15 (4.6) | 1.17 (0.40-3.40) | 0.29 (0.06-1.42) |
| **Education attainment** |  |  |  |  |  |
| No formal education | 84 | 32 (27.6) | 52 (16.0) | 1.00 | 1.00 |
| Primary education | 255 | 62 (53.4) | 193 (59.4) | **0.52 (0.31-0.88)** | **0.51 (0.29-0.92)** |
| Secondary education | 62 | 15 (12.9) | 47 (14.5) | 0.52 (0.25-1.08) | 0.49 (0.21-1.18) |
| High school | 12 | 0 (0.0) | 12 (3.7) | 1.00 (-----------) | 1.00 (------------) |
| Tertiary/university | 28 | 7 (6.0) | 21 (6.5) | 0.54 (0.21-1.42) | 0.44 (0.14-1.32) |

*Adjusted for the socio-demographics on the table.

**Table S1 (d): Belief that cervical cancer is curable only with traditional medicines**

| **Patient socio-demographic characteristics** | **Population responding** | **Curable only with traditional medicines** | | **Crude OR (COR) (95% CI)** | **Adjusted OR (AOR) (95%CI)*** |
| --- | --- | --- | --- | --- | --- |
|  |  | **Yes** | **No** |  |  |
|  |  | **N (%)** | **N (%)** |  |  |
| **Residence** |  |  |  |  |  |
| Urban | 80 | 4 (16.7) | 76 (20.8) | 1.00 | 1.00 |
| Rural | 310 | 20 (83.3) | 290 (79.2) | 1.31 (0.43-3.95) | 1.21 (0.33-4.39) |
| **Sex** |  |  |  |  |  |
| Women | 293 | 16 (66.7) | 277 (75.7) | 1.00 | 1.00 |
| Men | 97 | 8 (33.3) | 89 (24.3) | 1.56 (0.64-3.76) | 2.97 (0.87 -10.1) |
| **Age group (years)** |  |  |  |  |  |
| 18 - 29 | 145 | 7 (29.2) | 138 (37.8) | 1.00 | 1.00 |
| 30 - 44 | 139 | 2 (8.3) | 137 (37.5) | 0.29 (0.06-1.41) | **0.13 (0.02-0.94)** |
| 45 - 59 | 59 | 4 (16.7) | 55 (15.1) | 1.43 (0.40-5.09) | 0.56 (0.08-3.71) |
| ≥ 60 | 46 | 11 (45.8) | 35 (9.6) | **6.20 (2.24-17.14)** | 1.80 (0.28-11.44) |
| **Marital status** |  |  |  |  |  |
| Never Married | 21 | 1 (4.2) | 20 (5.5) | 1.00 | 1.00 |
| Married | 279 | 14 (58.3) | 265 (72.4) | 1.06 (0.13-8.45) | 1.26 (0.06-28.2) |
| Others | 90 | 9 (37.5) | 81 (22.1) | 2.22 (0.27-18.57) | 1.31 (0.04-43.28) |
| **Number of biological children** |  |  |  |  |  |
| None | 30 | 2 (8.3) | 28 (7.7) | 1.00 | 1.00 |
| 1 - 4 | 170 | 8 (33.3) | 162 (44.5) | 0.69 (0.14 -3.43) | 0.81 (0.08-8.10) |
| 5 - 10 | 165 | 12 (50.0) | 153 (42.0) | 1.10 (0.23-5.17) | 1.39 (0.14-13.59) |
| 11+ | 23 | 2 (8.3) | 21 (5.8) | 1.33 (0.17-10.25) | 0.79 (0.05-11.73) |
| **Education attainment** |  |  |  |  |  |
| No formal education | 68 | 11 (45.8) | 57 (15.6) | 1.00 | 1.00 |
| Primary education | 231 | 10 (41.7) | 221 (60.5) | **0.23 (0.09-0.58)** | **0.22 (0.07-0.71)** |
| Secondary education | 53 | 1 (4.2) | 52 (14.2) | **0.10 (0.01-0.80)** | **0.08 (0.01-0.90)** |
| High school | 12 | 0 (0.0) | 12 (3.3) | 1.00 (------------) | 1.00 (------------) |
| Tertiary/university | 25 | 2 (8.3) | 23 (6.3) | 0.45 (0.09-2.19) | 0.25 (0.03-2.00) |

*Adjusted for the socio-demographics on the table.

**Table S2 (a): Have often heard about cervical cancer through the local FM radio**

| **Patient socio-demographic characteristics** | **Population responding** | **Have heard about cervical cancer from local FM radios** | | **Crude OR (COR) (95% CI)** | **Adjusted OR (AOR) (95%CI)*** |
| --- | --- | --- | --- | --- | --- |
|  |  | **Yes** | **No** |  |  |
|  |  | **N (%)** | **N (%)** |  |  |
| **Residence** |  |  |  |  |  |
| Urban | 89 | 71 (23.1) | 18 (13.7) | 1.00 | 1.00 |
| Rural | 349 | 236 (76.9) | 113 (86.3) | **0.53 (0.30-0.93)** | **0.50 (0.27-0.93)** |
| **Sex** |  |  |  |  |  |
| Women | 332 | 223 (72.6) | 109 (83.2) | 1.00 | 1.00 |
| Men | 106 | 84 (27.4) | 22 (16.8) | **1.87 (1.11-3.15)** | 1.60 (0.88-2.90) |
| **Age group (years)** |  |  |  |  |  |
| 18 - 29 | 168 | 122 (39.7) | 46 (35.4) | 1.00 | 1.00 |
| 30 - 44 | 153 | 111 (36.2) | 42 (32.3) | 1.00 (0.61-1.63) | 0.85 (0.44-1.63) |
| 45 - 59 | 69 | 46 (15.0) | 23 (17.7) | 0.75 (0.41-1.38) | 0.74 (0.33-1.62) |
| ≥ 60 | 47 | 28 (9.1) | 19 (14.6) | 0.56 (0.28-1.09) | 0.59 (0.22-1.57) |
| **Marital status** |  |  |  |  |  |
| Never Married | 23 | 17 (5.5) | 6 (4.6) | 1.00 | 1.00 |
| Married | 314 | 227 (73.9) | 87 (66.4) | 0.92 (0.35-2.41) | 1.14 (0.26-5.04) |
| Others | 101 | 63 (20.5) | 38 (29.0) | 0.59 (0.21-1.61) | 0.96 (0.19-4.83) |
| **Number of biological children** |  |  |  |  |  |
| None | 33 | 22 (7.2) | 11 (8.4) | 1.00 | 1.00 |
| 1 - 4 | 197 | 138 (45.2) | 59 (45.0) | 1.17 (0.53-2.56) | 1.19 (0.35-4.03) |
| 5 - 10 | 181 | 127 (41.6) | 54 (41.2) | 1.18 (0.53-2.59) | 1.79 (0.49-6.46) |
| 11+ | 25 | 18 (2.9) | 7 (5.3) | 1.29 (0.41-4.00) | 2.06 (0.44-9.66) |
| **Education attainment** |  |  |  |  |  |
| No formal education | 83 | 46 (15.0) | 37 (28.5) | 1.00 | 1.00 |
| Primary education | 254 | 184 (60.0) | 70 (53.8) | **2.11 (1.27-3.53)** | **1.89 (1.07-3.34)** |
| Secondary education | 62 | 47 (15.3) | 15 (11.5) | **2.52 (1.22-5.20)** | 1.87 (0.80-4.39) |
| High school | 11 | 8 (2.6) | 3 (2.3) | 2.14 (0.53-8.66) | 1.09 (0.23-5.18) |
| Tertiary/university | 27 | 22 (7.2) | 5 (3.8) | **3.54 (1.22-10.25)** | 2.52 (0.78-8.13) |

*Adjusted for the socio-demographics on the table.

**Table S2 (b): Have often heard about cervical cancer from healthcare professionals**

| **Patient socio-demographic characteristics** | **Population responding** | **Heard about cervical cancer from health professionals** | | **Crude OR (COR) (95% CI)** | **Adjusted OR (AOR) (95%CI)*** |
| --- | --- | --- | --- | --- | --- |
|  |  | **Yes** | **No** |  |  |
|  |  | **N (%)** | **N (%)** |  |  |
| **Residence** |  |  |  |  |  |
| Urban | 93 | 26 (19.3) | 67 (22.9) | 1.00 | 1.00 |
| Rural | 335 | 109 (80.7) | 226 (77.1) | 1.24 (0.75-2.06) | 1.20 (0.67-2.14) |
| **Sex** |  |  |  |  |  |
| Women | 328 | 114 (84.4) | 214 (73.0) | 1.00 | 1.00 |
| Men | 100 | 21 (15.6) | 79 (27.0) | **0.50 (0.29-0.85)** | **0.47 (0.26-0.86)** |
| **Age group (years)** |  |  |  |  |  |
| 18 - 29 | 166 | 46 (34.3) | 120 (41.0) | 1.00 | 1.00 |
| 30 - 44 | 149 | 58 (43.3) | 91 (31.0) | **1.66 (1.04-2.67)** | **2.40 (1.26-4.57)** |
| 45 - 59 | 66 | 24 (12.9) | 42 (14.3) | 1.49 (0.81-2.73) | **2.33 (1.06-5.12)** |
| ≥ 60 | 46 | 6 (4.5) | 40 (13.7) | **0.39 (0.16-0.98)** | 0.86 (0.27-2.70) |
| **Marital status** |  |  |  |  |  |
| Never Married | 22 | 5 (3.7) | 17 (5.8) | 1.00 | 1.00 |
| Married | 306 | 102 (75.6) | 204 (69.6) | 1.70 (0.61-4.74) | 0.68 (0.14-3.24) |
| Others | 100 | 28 (20.7) | 72 (24.6) | 1.32 (0.45-3.93) | 0.43 (0.08-2.36) |
| **Number of biological children** |  |  |  |  |  |
| None | 33 | 7 (5.2) | 26 (8.9) | 1.00 | 1.00 |
| 1 - 4 | 196 | 63 (47.0) | 133 (45.4) | 1.76 (0.72-4.27) | 1.60 (0.43-5.94) |
| 5 - 10 | 174 | 59 (44.0) | 115 (39.2) | 1.91 (0.78-4.65) | 1.09 (0.27-4.32) |
| 11+ | 24 | 5 (3.7) | 19 (6.5) | 0.98 (0.27-3.55) | 0.69 (0.13-3.77) |
| **Education attainment** |  |  |  |  |  |
| No formal education | 79 | 26 (19.2) | 53 (18.2) | 1.00 | 1.00 |
| Primary education | 252 | 83 (61.5) | 169 (57.9) | 1.00 (0.58-1.71) | 1.05 (0.58-1.91) |
| Secondary education | 59 | 18 (13.3) | 41 (14.0) | 0.89 (0.43-1.85) | 1.12 (0.48-2.64) |
| High school | 10 | 4 (3.0) | 6 (2.1) | 1.36 (0.35-5.24) | 1.17 (0.39-7.57) |
| Tertiary/university | 27 | 4 (3.0) | 23 (7.9) | 0.35 (0.11-1.13) | 0.47 (0.13-1.69) |

*Adjusted for the socio-demographics on the table.

**Table S3 (a): Awareness that cervical cancer is preventable**

| **Patient socio-demographic characteristics** | **Population responding** | **Cervical cancer is Preventable** | | **Crude OR (COR) (95% CI)** | **Adjusted OR (AOR) (95%CI)*** |
| --- | --- | --- | --- | --- | --- |
|  |  | **Yes** | **No** |  |  |
|  |  | **N (%)** | **N (%)** |  |  |
| **Residence** |  |  |  |  |  |
| Urban | 93 | 72 (23.1) | 21(15.9) | 1.00 | 1.00 |
| Rural | 351 | 240 (76.9) | 111 (84.1) | 0.63 (0.37-1.08) | 0.78 (0.43-1.40) |
| **Sex** |  |  |  |  |  |
| Women | 336 | 232 (74.4) | 104 (78.8) | 1.00 | 1.00 |
| Men | 108 | 80 (25.6) | 28 (21.2) | 1.28 (0.79-2.09) | 1.09 (0.61-1.95) |
| **Age group (years)** |  |  |  |  |  |
| 18 - 29 | 171 | 132 (42.3) | 39 (29.8) | 1.00 | 1.00 |
| 30 - 44 | 155 | 109 (34.9) | 46 (35.1) | 0.70 (0.43-1.15) | 0.93 (0.48-1.82) |
| 45 - 59 | 70 | 46 (14.7) | 24 (18.3) | 0.57 (0.31-1.04) | 0.92 (0.41-2.03) |
| ≥ 60 | 47 | 25 (8.0) | 22 (16.8) | **0.34 (0.17-0.66)** | 0.58 (0.22-1.51) |
| **Marital status** |  |  |  |  |  |
| Never Married | 23 | 20 (6.4) | 3 (2.3) | 1.00 | 1.00 |
| Married | 318 | 226 (72.4) | 92 (69.7) | 0.37 (0.11-1.27) | 0.80 (0.14-4.57) |
| Others | 103 | 66 (21.2) | 37 (28.0) | **0.27 (0.07-0.96)** | 0.84 (0.13-5.43) |
| **Number of biological children** |  |  |  |  |  |
| None | 33 | 27(8.7) | 6 (4.5) | 1.00 | 1.00 |
| 1 - 4 | 201 | 149 (48.1) | 52 (39.4) | 0.64 (0.25-1.63) | 0.62 (0.15-2.50) |
| 5 - 10 | 183 | 120 (38.7) | 63 (47.7) | 0.42 (0.17-1.08) | 0.53 (0.13-2.23) |
| 11+ | 25 | 14 (4.5) | 11 (8.3) | **0.28 (0.09-0.93)** | 0.37 (0.07-1.91) |
| **Education attainment** |  |  |  |  |  |
| No formal education | 84 | 44 (14.1) | 40 (30.5) | 1.00 | 1.00 |
| Primary education | 257 | 190 (60.9) | 67 (51.1) | **2.58 (1.55-4.30)** | **2.32(1.33-4.05)** |
| Secondary education | 62 | 44 (14.1) | 18 (13.7) | **2.22 (1.12-4.46)** | 1.54 (0.68-3.52) |
| High school | 12 | 12 (3.8) | 0 (0.0) | 1.00 (------------) | 1.00 (------------) |
| Tertiary/university | 28 | 22 (7.1) | 6 (4.6) | **3.33 (1.23-9.05)** | 2.31 (0.76-7.01) |

*Adjusted for the socio-demographics on the table.

**Table S3 (b): Awareness that cervical cancer is curable in hospital when diagnosed in early stage**

| **Patient socio-demographic characteristics** | **Population responding** | **Cervical cancer curable if detected in early stage** | | **Crude OR (COR) (95% CI)** | **Adjusted OR (AOR) (95%CI)*** |
| --- | --- | --- | --- | --- | --- |
|  |  | **Yes** | **No** |  |  |
|  |  | **N (%)** | **N (%)** |  |  |
| **Residence** |  |  |  |  |  |
| Urban | 85 | 77 (20.9) | 8 (17.8) | 1.00 | 1.00 |
| Rural | 329 | 292 (79.1) | 37 (82.2) | 0.82 (0.37-1.83) | 0.94 (0.39-2.28) |
| **Sex** |  |  |  |  |  |
| Women | 314 | 277 (75.1) | 37 (82.2) | 1.00 | 1.00 |
| Men | 100 | 92 (24.9) | 8 (17.8) | 1.54 (0.69-3.42) | 1.23 (0.49-3.07) |
| **Age group (years)** |  |  |  |  |  |
| 18 - 29 | 163 | 145 (39.4) | 18 (40.0) | 1.00 | 1.00 |
| 30 - 44 | 146 | 133 (36.1) | 13 (28.9) | 1.27 (0.60-2.69) | 1.17 (0.43-3.18) |
| 45 - 59 | 65 | 57 (15.5) | 8 (17.8) | 0.88 (0.36-2.15) | 1.05 (0.33-3.34) |
| ≥ 60 | 39 | 33 (9.0) | 6 (13.3) | 0.68 (0.25-1.85) | 1.05 (0.25-4.43) |
| **Marital status** |  |  |  |  |  |
| Never Married | 21 | 19 (5.1) | 2 (4.4) | 1.00 | 1.00 |
| Married | 299 | 269 (72.9) | 30 (66.7) | 0.94 (0.21-4.25) | 0.55 (0.07-4.47) |
| Others | 94 | 81 (22.0) | 13 (28.9) | 0.66 (0.14-3.15) | 0.43 (0.04-4.25) |
| **Number of biological children** |  |  |  |  |  |
| None | 29 | 24 (6.5) | 5 (11.1) | 1.00 | 1.00 |
| 1 - 4 | 191 | 170 (46.3) | 21 (46.7) | 1.69 (0.58-4.89) | 2.22 (0.46-10.76) |
| 5 - 10 | 168 | 153 (41.7) | 15 (33.3) | 2.13 (0.71-6.38) | 3.14 (0.56-17.54) |
| 11+ | 24 | 20 (5.4) | 4 (8.9) | 1.04 (0.25-4.41) | 1.54 (0.21-11.18) |
| **Education attainment** |  |  |  |  |  |
| No formal education | 72 | 61 (16.5) | 11 (25.0) | 1.00 | 1.00 |
| Primary education | 241 | 216 (58.5) | 25 (56.8) | 1.56 (0.73-3.34) | 1.55 (0.67-3.54) |
| Secondary education | 60 | 54 (14.6) | 6 (13.6) | 1.62 (0.56-4.68) | 1.64 (0.47-5.67) |
| High school | 12 | 12 (3.3) | 0 (0.0) | 1.00 (-------------) | 1.00 (-------------) |
| Tertiary/University | 28 | 26 (7.0) | 2 (4.5) | 2.34 (0.49-11.32) | 2.47 (0.43-14.24) |

*Adjusted for the socio-demographics on the table.

**Table S4 (a): Recognized early onset of sexual intercourse as risk factor for cervical cancer**

| **Patient socio-demographic characteristics** | **Population responding** | **Recognized early onset of intercourse as risk factor** | | **Crude OR (COR) (95% CI)** | **Adjusted OR (AOR) (95%CI)*** |
| --- | --- | --- | --- | --- | --- |
|  |  | **Yes** | **No** |  |  |
|  |  | **N (%)** | **N (%)** |  |  |
| **Residence** |  |  |  |  |  |
| Urban | 90 | 11 (15.5) | 79 (24.7) | 1.00 | 1.00 |
| Rural | 301 | 60 (84.5) | 241 (75.3) | 1.79 (0.90-3.57) | 1.55 (0.72-3.34) |
| **Sex** |  |  |  |  |  |
| Women | 301 | 60 (84.5) | 241 (75.3) | 1.00 | 1.00 |
| Men | 90 | 11 (15.5) | 79 (24.7) | 0.56 (0.28-1.12) | 0.43 (0.37-2.63) |
| **Age group (years)** |  |  |  |  |  |
| 18 - 29 | 154 | 29 (40.8) | 125 (39.2) | 1.00 | 1.00 |
| 30 - 44 | 136 | 19 (26.8) | 117 (36.7) | 0.70 (0.37-1.32) | 0.43 (0.17-1.08) |
| 45 - 59 | 62 | 17 (23.9) | 45 (14.1) | 1.63 (0.82-3.24) | 0.99 (0.37-2.63) |
| ≥ 60 | 38 | 6 (8.5) | 32 (10.0) | 0.81 (0.31-2.11) | 0.65 (0.17-2.41) |
| **Marital status** |  |  |  |  |  |
| Never Married | 19 | 4 (5.6) | 15 (4.7) | 1.00 | 1.00 |
| Married | 281 | 54 (76.1) | 227 (70.9) | 0.89 (0.28-2.80) | 0.70 (0.12-4.24) |
| Others | 91 | 13 (18.3) | 78 (24.4) | 0.63 (0.18-2.18) | 0.44 (0.06-3.26) |
| **Number of biological children** |  |  |  |  |  |
| None | 30 | 6 (8.5) | 24 (7.5) | 1.00 | 1.00 |
| 1 - 4 | 176 | 25 (35.2) | 151 (47.2) | 0.66 (0.25-1.78) | 0.82 (0.19-3.61) |
| 5 - 10 | 163 | 34 (47.9) | 129 (40.3) | 1.05 (0.40-2.78) | 1.96 (0.40-9.52) |
| 11+ | 22 | 6 (8.5) | 16 (5.0) | 1.50 (0.41-5.48) | 2.34 (0.37-14.75) |
| **Education attainment** |  |  |  |  |  |
| No formal education | 72 | 15 (21.1) | 57 (17.8) | 1.00 | 1.00 |
| Primary education | 229 | 42 (59.2) | 187 (58.4) | 0.85 (0.44-1.65) | 0.96 (0.47-1.98) |
| Secondary education | 54 | 9 (12.7) | 45 (14.1) | 0.76 (0.30-1.90) | 1.23 (0.42-3.57) |
| High school | 11 | 0 (0.0) | 11 (3.4) | 1.00 (------------) | 1.00 (------------) |
| Tertiary/university | 25 | 5 (7.0) | 20 (6.3) | 0.95 (0.31-2.95) | 1.79 (0.49-6.55) |

*Adjusted for the socio-demographics on the table.

**Table 8 (b): Recognized multiple male sexual partners as risk factor for cervical cancer**

| **Patient socio-demographic characteristics** | **Population responding** | **Recognised multiple male partners as risk factor** | | **Crude OR (COR) (95% CI)** | **Adjusted OR (AOR) (95%CI)*** |
| --- | --- | --- | --- | --- | --- |
|  |  | **Yes** | **No** |  |  |
|  |  | **N (%)** | **N (%)** |  |  |
| **Residence** |  |  |  |  |  |
| Urban | 93 | 88 (23.3) | 5 (11.1) | 1.00 | 1.00 |
| Rural | 330 | 290 (76.7) | 40 (88.9) | 0.41 (0.16-1.08) | 0.54 (0.19-1.50) |
| **Sex** |  |  |  |  |  |
| Women | 321 | 289 (76.5) | 32 (71.1) | 1.00 | 1.00 |
| Men | 102 | 89 (23.5) | 13 (28.9) | 0.76 (0.38-1.51) | 0.73 (0.33-1.61) |
| **Age group (years)** |  |  |  |  |  |
| 18 - 29 | 162 | 143 (37.9) | 19 (42.2) | 1.00 | 1.00 |
| 30 - 44 | 147 | 134 (35.5) | 13 (28.9) | 1.37 (0.65-2.88) | 1.33 (0.49-3.64) |
| 45 - 59 | 66 | 57 (15.1 | 9 (20.0) | 0.84 (0.36-1.97) | 0.81 (0.26-2.54) |
| ≥ 60 | 47 | 43 (11.4) | 4 (8.9) | 1.43 (0.46-4.43) | 1.44 (0.28-7.48) |
| **Marital status** |  |  |  |  |  |
| Never Married | 22 | 19 (5.0) | 3 (6.7) | 1.00 | 1.00 |
| Married | 301 | 265 (70.1) | 36 (80.0) | 1.16 (0.33-4.12) | 1.53 (0.15-15.37) |
| Others | 100 | 94 (24.9) | 6 (13.3) | 2.47 (0.57-10.77) | 3.45 (0.26-46.17) |
| **Number of biological children** |  |  |  |  |  |
| None | 33 | 28 (7.4) | 5 (11.1) | 1.00 | 1.00 |
| 1 - 4 | 191 | 172 (45.7) | 19 (42.2) | 1.62 (0.56-4.68) | 0.87 (0.12-6.41) |
| 5 - 10 | 172 | 154 (41.0) | 18 (40.0) | 1.53 (0.52-4.45) | 0.86 (0.11-6.93) |
| 11+ | 25 | 22 (5.9) | 3 (6.7) | 1.31 (0.28-6.09) | 0.89 (0.08-9.93) |
| **Education attainment** |  |  |  |  |  |
| No formal education | 78 | 71 (18.8) | 7 (15.9) | 1.00 | 1.00 |
| Primary education | 248 | 218 (57.7) | 30 (68.2) | 0.72 (0.30-1.70) | 0.89 (0.35-2.28) |
| Secondary education | 58 | 53 (14.0) | 5 (11.4) | 1.05 (0.31-3.48) | 1.38 (0.35-5.369) |
| High School | 11 | 11 (2.9) | 0 (0.0) | 1.00 (------------) | 1.00 (------------) |
| Tertiary/university | 27 | 25 (6.6) | 2 (4.5) | 1.23 (0.24-6.33) | 1.42 (0.23-8.81) |

*Adjusted for the socio-demographics on the table.

**Table 8 (c): Recognized multi-parity as risk factor for cervical cancer**

| **Patient socio-demographic characteristics** | **Population responding** | **Recognized multi-parity as risk factor** | | **Crude OR (COR) (95% CI)** | **Adjusted OR (AOR) (95%CI)*** |
| --- | --- | --- | --- | --- | --- |
|  |  | **Yes** | **No** |  |  |
|  |  | **N (%)** | **N (%)** |  |  |
| **Residence** |  |  |  |  |  |
| Urban | 86 | 50 (25.6) | 36 (19.9) | 1.00 | 1.00 |
| Rural | 290 | 145 (74.4) | 145 (80.1) | 0.72 (0.44-1.17) | 0.17 (0.14-1.22) |
| **Sex** |  |  |  |  |  |
| Women | 286 | 147 (75.4) | 139 (76.8) | 1.00 | 1.00 |
| Men | 90 | 48 (24.6) | 42 (23.2) | 1.08 (0.67-1.74) | 1.18 (0.68-2.04) |
| **Age group (years)** |  |  |  |  |  |
| 18 - 29 | 143 | 84 (43.3) | 59 (32.6) | 1.00 | 1.00 |
| 30 - 44 | 133 | 70 (36.1) | 63 (34.8) | 0.78 (0.48-1.26) | 0.85 (0.45-1.61) |
| 45 - 59 | 61 | 28 (14.4) | 33 (18.2) | 0.60 (0.32-1.09) | 0.67 (0.31-1.43) |
| ≥ 60 | 38 | 12 (6.2) | 26 (14.4) | **0.32 (0.15-0.69)** | 0.38 (0.14-1.04) |
| **Marital status** |  |  |  |  |  |
| Never Married | 21 | 12 (6.2) | 9 (4.7) | 1.00 | 1.00 |
| Married | 265 | 143 (73.3) | 122 (67.4) | 0.88 (0.36-2.16) | 0.76 (0.18-3.30) |
| Others | 90 | 40 (20.5) | 50 (27.6) | 0.60 (0.23-1.57) | 0.64 (0.13-3.18) |
| **Number of biological children** |  |  |  |  |  |
| None | 32 | 16 (8.2) | 16 (8.8) | 1.00 | 1.00 |
| 1 - 4 | 166 | 95 (48.7) | 71 (39.2) | 1.34 (0.63-2.86) | 1.61 (0.47-5.52) |
| 5 - 10 | 155 | 74 (37.9) | 81 (44.8) | 0.91 (0.43-1.96) | 1.38 (0.37-5.07) |
| 11+ | 23 | 10 (5.1) | 13 (7.2) | 0.77 (0.26-2.26) | 1.50 (0.33-6.96) |
| **Education attainment** |  |  |  |  |  |
| No formal education | 65 | 30 (15.4) | 35 (19.4) | 1.00 | 1.00 |
| Primary education | 223 | 116 (59.5) | 107 (59.4) | 1.26 (0.73-2.20) | 0.96 (0.53-1.75) |
| Secondary education | 51 | 31 (15.9) | 20 (11.1) | 1.81 (0.86-3.81) | 1.07 (0.45-2.52) |
| High school | 9 | 4 (2.0) | 5 (2.8) | 0.93 (0.23-3.79) | 0.47 (0.10-2.11) |
| Tertiary/university | 27 | 14 (7.2) | 13 (7.2) | 1.26 (0.51-3.09) | 0.79 (0.28-2.21) |

*Adjusted for the socio-demographics on the table.

**Table 8 (d): Recognized genital infection by a virus as risk factor for cervical cancer**

| **Patient socio-demographic characteristics** | **Population responding** | **Recognised genital viral infection as risk factor** | | **Crude OR (COR) (95% CI)** | **Adjusted OR (AOR) (95%CI)*** |
| --- | --- | --- | --- | --- | --- |
|  |  | **Yes** | **No** |  |  |
|  |  | **N (%)** | **N (%)** |  |  |
| **Residence** |  |  |  |  |  |
| Urban | 86 | 78 (22.8) | 8 (15.7) | 1.00 | 1.00 |
| Rural | 307 | 264 (77.2) | 43 (84.3) | 0.63 (0.28-1.40) | 0.73 (0.30-1.75) |
| **Sex** |  |  |  |  |  |
| Women | 301 | 260 (76.0) | 41 (80.4) | 1.00 | 1.00 |
| Men | 92 | 82 (24.0) | 10 (19.6) | 1.29 (0.62-2.70) | 1.04 (0.45-2.43) |
| **Age group (years)** |  |  |  |  |  |
| 18 - 29 | 152 | 133 (39.0) | 19 (37.3) | 1.00 | 1.00 |
| 30 - 44 | 136 | 119 (34.9) | 17 (33.3) | 1.00 (0.50-2.01) | 0.56 (0.22-1.39) |
| 45-59 | 63 | 53 (15.5) | 10 (19.6) | 0.76 (0.33-1.76) | 0.45 (0.16-1.31) |
| ≥ 60 | 41 | 36 (10.6) | 5 (9.8) | 1.03 (0.36-2.94) | 0.74 (0.16-3.36) |
| **Marital status** |  |  |  |  |  |
| Never Married | 21 | 19 (5.6) | 2 (3.9) | 1.00 | 1.00 |
| Married | 280 | 240 (70.2) | 40 (78.4) | 0.63 (0.14-2.82) | 0.79 (0.09-6.65) |
| Others | 92 | 83 (24.3) | 9 (17.6) | 0.97 (0.19-4.86) | 1.81 (0.17-19.68) |
| **Number of biological children** |  |  |  |  |  |
| None | 32 | 27 (7.9) | 5 (9.8) | 1.00 | 1.00 |
| 1 - 4 | 175 | 149 (43.6) | 26 (51.0) | 1.06 (0.37-3.01) | 1.04 (0.21-5.15) |
| 5 - 10 | 164 | 146 (42.7) | 18 (35.3) | 1.50 (0.51-4.39) | 2.70 (0.50-14.47) |
| 11+ | 22 | 20 (5.8) | 2 (3.9) | 1.85 (0.33-10.54) | 3.59 (0.40-32.02) |
| **Education attainment** |  |  |  |  |  |
| No formal education | 68 | 53 (15.5) | 15 (30.0) | 1.00 | 1.00 |
| Primary education | 235 | 208 (61.1) | 27 (54.0) | **2.18 (1.08-4.39)** | **2.51 (1.14-5.49)** |
| Secondary education | 53 | 48 (14.0) | 5 (10.0) | 2.72 (0.92-8.04) | **3.78 (1.09-13.16)** |
| High school | 9 | 9 (2.6) | 0 (0.0) | 1.00 (------------) | 1.00 (-------------) |
| Tertiary/university | 27 | 24 (7.0) | 3 (6.0) | 2.26 (0.60-8.56) | 2.90 (0.64-13.16) |

*Adjusted for the socio-demographics on the table.

**Table 9: Reports contraceptives use as risk factor for cervical cancer**

| **Patient socio-demographic characteristics** | **Population responding** | **Reports contraceptives use as risk factor** | | **Crude OR (COR) (95% CI)** | **Adjusted OR (AOR) (95%CI)*** |
| --- | --- | --- | --- | --- | --- |
|  |  | **Yes** | **No** |  |  |
|  |  | **N (%)** | **N (%)** |  |  |
| **Residence** |  |  |  |  |  |
| Urban | 84 | 50 (19.5) | 34 (27.0) | 1.00 | 1.00 |
| Rural | 299 | 207 (80.5) | 92 (73.0) | 1.53 (0.93-2.52) | 1.24 (0.71-2.20) |
| **Sex** |  |  |  |  |  |
| Women | 290 | 195 (75.9) | 95 (75.4) | 1.00 | 1.00 |
| Men | 93 | 62 (24.1) | 31 (24.6) | 0.97 (0.59-1.60) | 0.69 (0.35-1.37) |
| **Age group (years)** |  |  |  |  |  |
| 18 - 29 | 148 | 98 (38.1) | 50 (39.7) | 1.00 | 1.00 |
| 30 - 44 | 134 | 88 (34.2) | 46 (36.5) | 0.98 (0.60-1.60) | 0.69 (0.35-1.37) |
| 45 - 59 | 60 | 41 (16.0) | 19 (15.1) | 1.10 (0.58-2.09) | 0.66 (0.28-1.53) |
| ≥ 60 | 41 | 30 (11.7) | 11 (8.7) | 1.39 (0.64-3.01) | 0.72 (0.25-2.09) |
| **Marital status** |  |  |  |  |  |
| Never Married | 21 | 15 (5.8) | 6 (4.8) | 1.00 | 1.00 |
| Married | 272 | 182 (70.8) | 90 (71.4) | 0.81 (0.30-2.15) | 1.83 (0.30-11.18) |
| Others | 90 | 60 (23.3) | 30 (23.8) | 0.80 (0.28-2.27) | 1.75 (0.25-12.14) |
| **Number of biological children** |  |  |  |  |  |
| None | 31 | 24 (9.3) | 7 (5.6) | 1.00 | 1.00 |
| 1 - 4 | 171 | 104 (40.5) | 67 (53.2) | 0.45 (0.18-1.11) | 0.35 (0.07-1.72) |
| 5 - 10 | 157 | 111 (43.2) | 46 (36.5) | 0.70 (0.28-1.75) | 0.57 (0.11-3.02) |
| 11+ | 24 | 18 (7.0) | 6 (4.8) | 0.88 (0.25-3.05) | 0.67 (0.10-4.42) |
| **Education attainment** |  |  |  |  |  |
| No formal education | 70 | 54 (21.1) | 16 (12.7) | 1.00 | 1.00 |
| Primary education | 223 | 149 (58.2) | 74 (58.7) | 0.60 (0.32-1.11) | 0.57 (0.30-1.12) |
| Secondary education | 56 | 33 (12.9) | 23 (18.3) | **0.43 (0.20-0.92)** | 0.46 (0.19-1.11) |
| High school | 8 | 1 (0.4) | 7 (5.6) | **0.04 (0.00-0.37)** | **0.05 (0.01-0.45)** |
| Tertiary/university | 25 | 19 (7.4) | 6 (4.8) | 0.94 (0.32-2.75) | 1.09 (0.33-3.54) |

*Adjusted for the socio-demographics on the table.
